# Learning Monologues at Bedtime Improves Sleep Quality in Actors and Non-Actors

**DOI:** 10.3390/ijerph19010011

**Published:** 2021-12-21

**Authors:** Francesca Conte, Oreste De Rosa, Benedetta Albinni, Daniele Mango, Alessia Coppola, Serena Malloggi, Davide Giangrande, Fiorenza Giganti, Giuseppe Barbato, Gianluca Ficca

**Affiliations:** 1 Department of Psychology, University of Campania L. Vanvitelli, Viale Ellittico 31, 81100 Caserta, Italy; oreste.derosa@unicampania.it (O.D.R.); benedetta.albinni@unicampania.it (B.A.); daniele.mango@libero.it (D.M.); alessiacoppola001@gmail.com (A.C.); davide.giangrande@studenti.unicampania.it (D.G.); giuseppe.barbato@unicampania.it (G.B.); gianluca.ficca@unicampania.it (G.F.); 2 Department Neurofarba, University of Firenze, Via di San Salvi 12, 50135 Firenze, Italy; serena.malloggi@unifi.it (S.M.); fiorenza.giganti@unifi.it (F.G.)

**Keywords:** ecological learning, sleep continuity, sleep stability, sleep organization, wake intensity

## Abstract

Several studies show that pre-sleep learning determines changes in subsequent sleep, including improvements of sleep quality. Our aims were to confirm this finding using a more ecological task (learning a theatrical monologue) and to investigate whether the effect is modulated by expertise. Using a mixed design, we compared polysomnographic recordings of baseline sleep (BL, 9-h TIB) to those of post-training sleep (TR, with the same TIB but preceded by the training session), in one group of actors (*N* = 11) and one of non-actors (*N* = 11). In both groups, TR appears reorganized and re-compacted by the learning session, as shown, among others, by a significant decrease of WASO%, awakenings, arousals, and state transitions and by a trend towards an increased number of complete cycles and total cycle time. Concerning memory performance, the number of synonyms produced was significantly higher in the morning relative to immediate recall. No between-groups differences emerged either for sleep or memory variables. Our data confirm pre-sleep learning’s beneficial effect on sleep quality in an ecological context. While expertise appears not to influence memory-related sleep mechanisms, results on morning recall support the recent view that sleep’s role in memory processes consists in trace “transformation” for adaptive purposes, rather than rote consolidation.

## 1. Introduction

The issue of the influence of wake on subsequent sleep has been classically addressed through Borbély’s model of sleep regulation [1]. According to this classical model, the timing of sleep and, partially, its characteristics (i.e., namely the amount of slow wave Sleep, SWS), may be predicted based on previous wake duration and its interplay with circadian factors. However, even before the formulation of the two-process model [1], Feinberg had proposed that sleep is also modulated by the *intensity* of waking brain activity [2], which was measured, in those pioneering studies, through brain temperature or cerebral metabolic rate [3,4,5]. This idea that wake quality rather than its mere duration bears significant effects on sleep has repeatedly reappeared in later years [6,7], with reference, for instance, to significant evidence of sleep changes (namely delta activity) in rats after behavioral manipulations [6,8].

Quite surprisingly, however, this issue has never been purposefully and systematically addressed in sleep literature. In other words, after Feinberg’s pioneering work, very few studies have specifically aimed to manipulate waking activity to assess subsequent sleep changes (e.g., [9,10,11,12]), despite the important implications of such an approach. A better understanding of the influence of wake intensity on sleep characteristics could contribute to refining existing models of sleep regulation and, from an applicative standpoint, could represent the basis for the construction of behavioral protocols aimed at manipulating waking activities in order to obtain desired changes in sleep [13,14]. 

A contribution in this direction has been provided in a recent review from our group [14], in which we overviewed studies documenting the presence of sleep changes after behavioral manipulations of waking cognitive activity. In addition to studies based on “enriched environment” procedures (mostly conducted on animal samples), we described the wide literature on post-learning sleep modifications coming from the field of research on sleep-memory relationships. Indeed, a common experimental approach in this research domain consists in comparing a baseline sleep episode with one preceded by a learning task: the modifications emerging in post-learning sleep characteristics are believed to reflect the involvement of such features in the overnight consolidation of the learned material. 

The results of our review highlighted that, although the first findings were limited to SWS rebounds (e.g., [3,6,8]), the range of sleep variables influenced by pre-sleep learning is actually much wider [14]. For instance, spindle parameters appear consistently enhanced (e.g., [15,16,17]). Furthermore, it is particularly interesting that also sleep continuity and stability measures, traditionally considered as markers of sleep quality, frequently show improvements after wake content manipulation (e.g., [9,10,11,12,18,19]). These studies point to the intriguing possibility to reduce sleep fragmentation and instability through planned cognitive and behavioral interventions.

Several possible directions for future research also emerged from our review [14]. One significant issue regards ecological validity: the data from enriched environment paradigms (e.g., [20,21]) support the hypothesis that relevant sleep changes are determined by wake intensity manipulations that do not necessarily include typical laboratory pre-sleep tasks. In this perspective, we have recently obtained promising results through a complex multi-componential task (similar to the well-known videogame Ruzzle), requiring, as in most everyday-life circumstances, the combined activation of several cognitive processes, including both basic functions such as procedural motor memory and executive functions. Its pre-sleep administration determined improvements in participants’ sleep propensity, continuity, and stability in a daytime nap [11] as well as in a night sleep episode [12]. Additionally, improvements in sleep cyclic organization were also observed in the latter study.

Another open question highlighted in our review [14] regards the numerous factors which could modulate the effects of waking cognitive processes on sleep (see also [13]). Clarifying this issue appears particularly important when planning targeted behavioral interventions for sleep improvement. Among these factors, task difficulty and expertise have repeatedly been shown to modulate sleep-related memory consolidation and therefore are likely to play a role in how waking cognitive activity influences sleep. In several studies, “good learners” showed either the greater post-sleep performance benefits [22,23] or more pronounced post-learning sleep modifications [24,25]. 

To address these issues, here, we compare a baseline sleep episode with one preceded by intensive training on a verbal ecological task (learning a theatrical monologue) in a group of professional actors and a control group of non-actors. We hypothesize that:

(a) Compared to baseline sleep, post-learning sleep will show enhancements of those sleep features that are involved in sleep-related memory consolidation (with special attention to sleep continuity, stability, and organization variables)

(b) These changes will appear more pronounced in the group of actors, who are assumed to be “experts” in this specific type of verbal prose learning by virtue of their profession. 

## 2. Materials and Methods

### 2.1. Participants

The group of actors was recruited through one of the experimenters’ personal contacts with a theater director. Potential participants for this group had to have at least five years of experience as professional actors in order to be included in the screening process. The latter was conducted through a brief ad hoc interview aimed to collect general demographic data (age, gender, professional status) and information on medical condition (including sleep disorder symptoms) and health habits, as well as through the administration of the Italian versions of the Pittsburgh Sleep Quality Index (PSQI [26]), the Beck Anxiety Inventory (BAI [27]) and the Beck Depression Inventory (BDI-II [27]). The interview and the administration of screening questionnaires were conducted by a trained psychologist (A.C.), who did not participate in the data collection phases of the research. 

Inclusion criteria were: age 18–45 years; absence of any relevant somatic or psychiatric disorder; absence of sleep disorder symptoms; no history of drug or alcohol abuse; having a regular sleep-wake pattern (including going to bed between 22:30 and 00:30); limited caffeine (no more than 150 mg caffeine per day, corresponding to about three cups of espresso or one cup of American coffee) and alcohol (no more than 250 mL per day, i.e., about a pint of standard beer, a full glass of wine, or a small liquor shot) consumption, having a PSQI score < 5 (indicating good sleep quality [28]), a BAI score ≤ 25 (indicating absence of anxiety symptoms [29]), and a BDI-II score ≤ 29 (indicating absence of depressive symptoms [30]). 

Control (non-actor) participants were recruited through social media and university websites, using the same screening process and inclusion criteria except for professional status. 

Twenty-one actors and twenty-one non-actors were initially recruited for a pilot study that aimed to select the appropriate monologue for the learning and re-test phases of the study (see Section 2.2). Eleven more actors (5 F, age range: 24–32 years, mean age: 26 ± 3.4) and eleven non-actors (6 F, age range: 22–30 years, mean age: 27 ± 2.9) agreed to participate in the main study and made up the final sample. None of the participants (either in the pilot or main study) received any payment or credit compensation for their participation. All subjects signed a consent form prior to participation in the study.

The study design was submitted to the Ethical Committee of the Department of Psychology, University of Campania “L. Vanvitelli”, which approved the research (code: 14/2017) and certified that the involvement of human participants was performed according to acceptable standards.

### 2.2. Learning Task

A pilot study was conducted in order to select the monologue to be employed as a learning task in the main study. Three monologues of comparable length were selected by a theater director from the play “Stratégie pour deux jambons: roman chochon” (Italian title: “Strategia per due prosciutti”) by Raymond Cousse [31]. These were administered to the pilot study participants (21 actors and 21 non-actors) who were requested to study each monologue for 30 min: each subject studied each of the 3 monologues (in balanced order between subjects) with a 2-days interval. After each study session, participants were instructed to answer 3 questions: (1) How difficult was the text? (0 to 4 scale, from “not at all” to “very difficult”); (2) Do you feel that 30 min were sufficient for learning? (0 to 4 scale, from “not at all” to “more than sufficient”); (3) How much do you feel you have accurately learned the text? (0 to 4 scale, from “not at all” to “very much”). Finally, the monologue “Manure” (made up of 216 words) was selected for the main study, based on the fact that it was rated as “slightly difficult” by actors and “moderately difficult” by non-actors, that the time allotted for learning was “sufficient” for actors and “just sufficient” for non-actors, and that perceived accuracy of learning was “quite enough” for actors and “a little” for non-actors. 

The rating method for the learning task (main study) was based on that of Spinnler and Tognoni’s prose memory task [32], which is commonly included in standard batteries for neuropsychological assessment. As in Spinnler and Tognoni [32], the text we used is composed of “target” and “satellite” words, which convey at recall a score of 3 and 1 points, respectively. “Target” words are defined as words which hold the sentence’s semantic gist, while “satellite” words are those that are semantically related to the first ones. Prior to the experimental sessions, two of the experimenters independently coded all semantic words of the selected monologue as “target” or “satellite” words. Initial concordance was 90% and discrepancies were solved through mutual agreement between the experimenters. 

The final rating method for performance yields a global “accuracy score” made up of: correctly recalled target words (3 points), correctly recalled satellite words (1 point, but satellite words score 0 if recalled in absence of the target word they are cued to), synonyms (0.5 points, at variance with Spinnler and Tognoni [32], who attribute a full point) and a penalty of −0.5 points attributed to “grammar” errors (i.e., gender or singular/plural inversions, changes to the tenses of verbs). Again, scoring of recall performance was performed independently by two experimenters (D.G. and S.M.), who solved all discrepancies through discussion and mutual agreement. These experimenters were blind to the study groups and were not involved in other phases of data collection.

### 2.3. Procedure

Each subject underwent three nights of sleep recording at home, with 4–7-day intervals between sessions. An adaptation night was followed by 2 experimental conditions, whose order was balanced across participants: (1) baseline sleep (BL); (2) post-training sleep (TR), i.e., a sleep episode preceded by a verbal ecological task. 

During the 3 days preceding each recording session, subjects were requested to keep sleep-wake schedules and daily activities as habitual as possible and to avoid, on recording days, any cognitively engaging activity (e.g., reading, studying, playing cards, etc.) beyond habit. Moreover, participants were specifically instructed to maintain their daily activities as similar possible between the 2 recording days. To control for these factors (including sufficient sleep duration, napping habits, physical activity, caffeine and alcohol consumption), on each of the 3 days preceding recordings subjects filled a sleep log and a short ad-hoc diary on daily activities. 

On the days scheduled for sleep recording, the experimenter arrived at the subject’s house approximately one hour before usual bedtime and proceeded to electrodes set-up. While in BL subjects went to bed immediately after that, in TR subjects performed the behavioral task just before bedtime: subjects were allotted 30 min to learn by themselves the theatrical monologue (provided in written form on a printed piece of paper) and were informed that an immediate and a delayed recall phase would follow. They then had 15 min to verbally recall all they could from the text as precisely as possible (immediate recall phase). The report was audio-recorded for later scoring. The printed monologue was returned to the experimenter just after the learning phase in order to ensure that participants did not further rehearse the text after the experimenter’s departure. Participants went to bed immediately after the immediate recall phase. 

For the learning, immediate recall, and delayed recall phases, a quiet room of the participant’s house was chosen. The windows and shutters were kept closed and artificial light was used in order to keep illumination constant across the 3 phases. 

Bedtime and awakening time were not pre-determined: participants were asked to maintain their regular sleep-wake habits in both conditions. 

To control for sleepiness and fatigue levels, the Karolinska Sleepiness Scale (KSS [33]) and a Visual Analogue Scale (VAS, 0 cm = not tired at all and 10 cm = very tired) for fatigue [34] were administered in both conditions immediately before lights off. In addition, in TR, the two scales were also completed before task administration.

Upon awakening, subjects completed the sleep log. In TR, the KSS, the VAS and a re-test session were administered 30 min after morning final awakening: participants were allotted 15 min to verbally report all they could remember from the monologue, as precisely as possible (delayed recall phase). Again, reports were recorded through an audio-recorder for later scoring. 

The same experimenter (D.M.) performed electrode montage, administered both the main learning task and control tests (KSS and VAS scales), and collected the audio-recorders for all participants. 

The whole experimental phase of the study was conducted between January 2016 and March 2018 (i.e., before the COVID-19 outbreak).

### 2.4. Sleep Recordings and Sleep Measures

Polysomnographic recordings were performed by recording 6 electroencephalographic (EEG) (F3-A2, F4-A1, C3-A2, C4-A1, O1-A2, O2-A1), 2 electrooculographic (LOC-A2, ROC-A1), and a bipolar submental electromyogram channel according to standard guidelines [35]. Data were acquired by means of a BluNet multichannel recording system (Ne.Ro SRL, Florence, Italy) at a sample rate of 200 Hz. Sleep recordings were band-passed (0.3–35 Hz) and then visually scored according to standard criteria [35] by an expert technician, B.A., who had not participated in other phases of data collection and was blind to the study groups and conditions. To verify scoring reliability, 10 randomly selected sleep recordings were also scored by another technician. Inter-rater agreement was 93%.

Classical sleep architecture variables considered in the study were: sleep onset latency (SOL), time in bed (TIB, i.e., total amount of time, in minutes, from lights off to final awakening), total sleep time (TST, i.e., total amount of time, in minutes, from the first appearance of N1 to final awakening), actual sleep time (AST, i.e., total time spent in sleep states, expressed in minutes), sleep stage proportions (percentages over AST), sleep efficiency (SE, i.e., percentage of AST over Time in Bed), and percentage of wake after sleep onset over TST (WASO%).

As in Conte et al. [9], objective sleep quality was evaluated through an additional set of variables assessing:

1. Sleep continuity: Total frequency of awakenings per hour of AST; frequency of brief (<4 epochs) and long (≥4 epochs) awakenings per hour of AST
2. Sleep stability: Frequency of arousals per hour of AST (here arousals are defined as all transitions to shallower NREM sleep stages and from REM sleep to N1); frequency of state transitions (defined as all transitions from one state to another) per hour of TST; frequency of “functional uncertainty periods” (FU periods; defined as periods in which a minimum of 3 state transitions follow one another with no longer than 1.5 min intervals) per hour of TST; percentage of total time spent in FU (TFU) over TST;
3. Sleep organization: Number of complete sleep cycles, defined as sequences of NREM and REM sleep (each lasting at least 10 min) not interrupted by periods of wake longer than 2 min (as in [9]); percentage of total time spent in cycles (TCT) over TST. A necessary methodological remark concerns our choice of an extremely conservative definition of the sleep cycle, based on the assumption that a non-marginal amount of each sleep state is required for the NREM-REM cycle to exert its role in sleep-dependent memory processes [36]. With such a definition, the duration of a sleep cycle is remarkably shorter, on average, than that obtained with the more common, less conservative, definitions.

### 2.5. Performance Measures

Memory performance at baseline (immediate recall) and at post-sleep re-test (delayed recall) was assessed through the following measures: (1) global “accuracy score” (correct words + synonyms − penalties; see Section 2.2), which ranges from 0 to 224; (2) “synonyms” (falsely recalled words having the same meaning as those of the original text); (3) “intrusions” (false recalls excluding synonyms); (4) “omissions” (number of semantic words that were not recalled); (5) “inversions” (inversions in word order); (6) “grammar” errors (i.e., gender or singular/plural inversions, changes to the tenses of verbs).

### 2.6. Data Analysis

A 2-way mixed ANOVA was performed on sleep variables and bedtime KSS and VAS fatigue scores with “Group” (actors vs. non-actors) as between-groups factor and “Condition” (BL vs. TR) as within-groups factor. The same ANOVA was conducted on performance measures, as well as on KSS and VAS fatigue scores collected before training and recall, with “Group” (actors vs. non-actors) as between-groups factor and “Recall phase” (immediate vs. delayed) as within-groups factor. In case of significance, η^2^ was used as a measure of effect size and the Tuckey test for post-hoc analysis.

Following statistical guidelines to correct for multiple testing without running a too high risk of Type II Error (see, e.g., [37]), we applied to sleep measures analyses an adapted Bonferroni procedure: the conventional alpha value (*p* ≤ 0.05) was divided by four, i.e., by the number of relevant sleep “dimensions” addressed in our research (“sleep classical measures”, “sleep continuity”, “sleep stability”, “sleep organization”). Therefore, significance was set at *p* ≤ 0.0125.

The alpha level was maintained at *p* ≤ 0.05 for the analyses concerning performance measures, sleepiness, and fatigue levels.

All analyses were conducted using JAMOVI 1.6.23 [38]. 

The data were analyzed by O.D.R., who had not participated in data collection. 

## 3. Results

### 3.1. Sleepiness and Fatigue

Sleepiness levels at bedtime (collected just before lights off) did not differ neither between conditions nor between groups (Condition: F_3_ = 2.01, *p* = 0.122, η^2^ = 0.030; Interaction: F_3_ = 1.97, *p* = 0.127, η^2^ = 0.030 Group: F_1_ = 2.86, *p* = 0.106, η^2^ = 0.042). The same negative results emerged for bedtime fatigue (Condition: F_3_ = 2.00; *p* = 0.123, η^2^ = 0.044; Interaction: F_3_ = 0.62, *p* = 0.601, η^2^ = 0.014; Group: F_1_ = 1.28, *p* = 0.272, η^2^ = 0.030).

Similarly, in TR, no recall phase, group, or interaction effects emerged for sleepiness levels reported before training and before the recall session in the two groups (Recall phase: F_3_ = 0.2.09, *p* = 0.111, η^2^ = 0.046; Interaction: F_3_ = 0.72, *p* = 0.542, η^2^ = 0.016; Group: F_1_ = 1.12, *p* = 0.302, η^2^ = 0.027). The same negative finding was observed for fatigue (Recall phase: F_3_ = 1.88, *p* = 0.143, η^2^ = 0.051; Interaction: F_3_ = 1.01, *p* = 0.396, η^2^ = 0.027; Group: F_1_ = 0.14, *p* = 0.706, η^2^ = 0.003).

### 3.2. Classical Sleep Variables

Table 1 displays results on classical sleep measures. Sleep latency showed a significant effect of Condition, as well as a trend to a significant effect of Group and to a significant interaction. Specifically, it decreased in TR (8.02 ± 5.27 min) compared to BL (16 ± 10.5 min), with actors showing shorter sleep latency in both conditions (mean difference = −6.95). Post-hoc analysis yielded significant differences between BL and TR for actors (*p* = 0.004) and non-actors (*p* < 0.001).

Stage 1 (%) showed a trend towards a significant effect of Group, with actors displaying less Stage 1 than non-actors (mean difference = −0.080).

An effect of Condition emerged for WASO% and Sleep Efficiency. In both cases, TR showed an increase of sleep quality, with lower proportion of WASO and higher Sleep Efficiency.

No other significant effects emerged.

### 3.3. Sleep Continuity 

**Figure 1:**
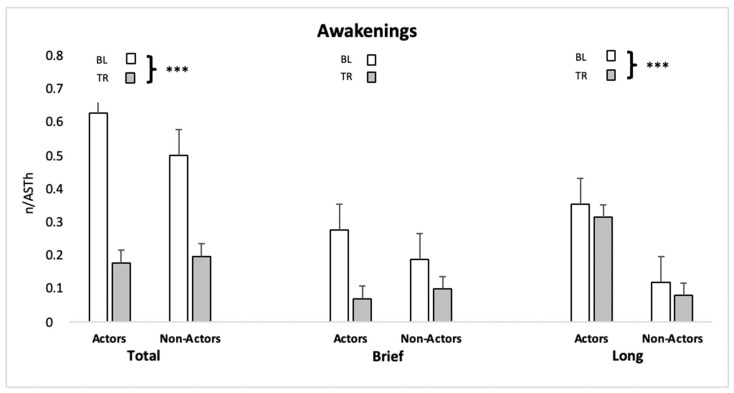
Frequency of total, brief and long behavioral awakenings in the two conditions in actors and non-actors. ***: *p* < 0.001.

Most sleep continuity variables showed a main effect of Condition whereas no Group nor Interaction effects emerged. Specifically, the total frequency of awakenings and frequency of long awakenings displayed a significant reduction in TR compared to BL (Figure 1, Table 2), whereas awakenings mean duration did not show any significant effect (Table 2). Brief awakenings, also displayed in Figure 1, showed a trend towards a significant effect of Condition, with a reduction in TR vs. BL. 

### 3.4. Sleep Stability 

**Figure 2:**
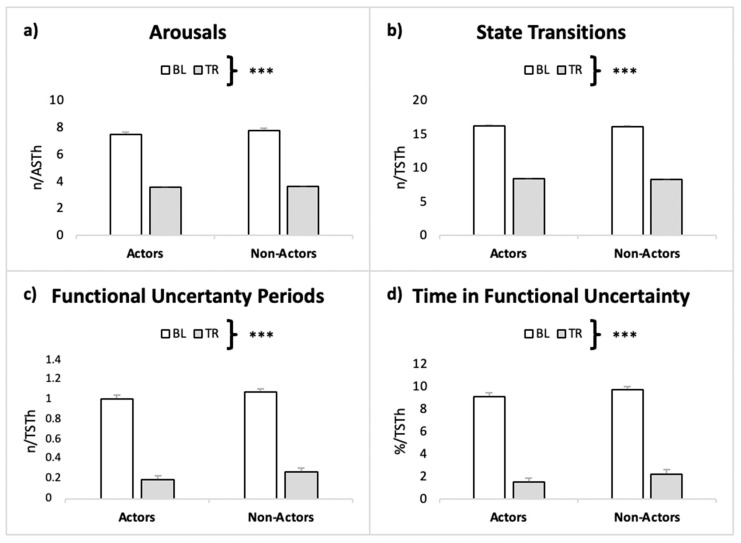
Sleep stability in the two conditions in actors and non-actors. (**a**) Frequency of arousals over Actual Sleep Time. (**b**) Frequency of state transitions over Total Sleep Time (TST). (**c**) Frequency of state transitions over TST. (**d**) Percentage of Time spent in Functional Uncertainty over TST. ***: *p* < 0.001.

All sleep stability measures showed a main effect of Condition with no Group or Interaction effects (Figure 2, Table 3).

### 3.5. Sleep Cyclic Organization 

**Figure 3:**
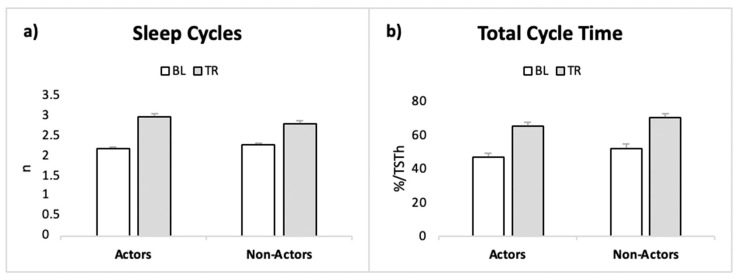
Sleep cyclic organization in the two conditions in actors and non-actors. (**a**) Number of sleep cycles. (**b**) Percentage of Total Cycle Time over Total Sleep Time.

Results on sleep organization are coherent with those on the other sleep measures. In fact, we observed a trend towards a main effect of Condition, indicating an increase in TR, for number of sleep cycles and Total cycle time %, with no Group or Interaction effects. Mean duration of sleep cycles displayed no significant effect (Figure 3, Table 4). 

### 3.6. Memory Performance

No performance measure displayed significant effects except for the number of synonyms, which showed a main effect of Condition (indicating an increase in TR compared to BL) and no Group or interaction effects (Table 5).

## 4. Discussion

Here, we aimed to explore the effects of a pre-sleep ecological task, i.e., learning a theatrical monologue, on subsequent sleep features, as well as to assess whether expertise in the task modulates these effects. To this end, we compared a baseline sleep episode with one preceded by a learning session in which participants rehearsed a theatrical monologue, in both a group of professional actors and one of non-actors. 

Consistent with our hypothesis, our main finding regards the robust effect of training on most sleep quality measures. In fact, in the whole sample, post-training sleep appeared re-compacted compared to baseline sleep, showing improved sleep continuity (higher sleep efficiency, lower WASO proportion, fewer awakenings) and stability (fewer arousals, state transitions and functional uncertainty periods, reduced time in functional uncertainty and reduced mean duration of functional uncertainty periods). Sleep cyclic organization also appeared to benefit from training (more numerous sleep cycles and increased proportion of time spent in cycles in TR), although the significance of these results did not survive Bonferroni’s correction. This pattern of findings confirms what we have previously observed after pre-sleep training in a word list task in a sample of elderly participants [9] and after training on a complex procedural–executive task in young adults both through a night-sleep paradigm [12] and through a nap paradigm [11]. Our findings are also in line with several other studies displaying improvements in sleep continuity, stability, and cyclic organization after pre-sleep cognitive tasks [10,18,19,39]. 

Furthermore, we extend these results to a task that is more ecological than those usually employed in laboratory studies. Indeed, among verbal tasks, those relying on prose memory are considered to have a higher resemblance to the cognitive processes required in everyday life [40]. This entails relevant theoretical and applicative implications. From a theoretical standpoint, it further supports the idea that a “process L” (“process Learning”, already proposed in [13,14]), expressed by the quality and quantity of daytime acquisition processes of everyday life, could be usefully included in an updated model of sleep regulation, along with the classical circadian and homeostatic processes [1]. In an applicative perspective, this supports the possibility to employ ecological protocols based on cognitively engaging activities in order to obtain improvements in sleep quality in populations with sleep impairments [14]. Encouraging results in this direction have already been obtained on healthy older adults [9], on older adults with insomnia [10], and on adult poor sleepers [12]. 

As a side remark, it is worth noting that our findings on sleep stability parameters, along with other data from clinical [41,42] and non-clinical studies [43], encourage one to consider the introduction of these measures in standard sleep assessments as additional indices of sleep quality. In fact, it has been suggested [12] that traditional sleep quality measures such as sleep efficiency could be insufficient to capture the dynamics of overnight disturbing events, as shown by several studies in which more fine-grained analyses have proven more useful than classical parameters to describe objective sleep quality [44,45]. Specifically, sleep stability measures (e.g., frequency of arousals and state transitions) have been proposed, along with sleep continuity indices, as markers of disturbed sleep [9,46]. 

An important theoretical issue regards the mechanisms sustaining the re-compacting effect on sleep observed in our sample. Our pattern of findings is consistent with the sequential hypothesis on sleep-related memory consolidation [36,47,48], which proposes that the interplay between NREM and REM states, rather than their absolute amount, is essential for the overnight consolidation of learned material. In fact, it is plausible that the improvements of sleep continuity, stability and organization emerged in our study reflect the involvement of such sleep parameters in the consolidation process. In this regard, a limitation of our study resides in the fact that we did not employ an active control condition (i.e., in which the sleep episode is preceded by a non-learning control task, as in [10,12,49,50]), which would have allowed us to disentangle learning-dependent from general use-dependent effects on sleep features. However, we have previously shown, in a sample of poor sleepers, that several sleep stability and organization measures were specifically affected by training rather than by a control task [12]. Therefore, although we cannot exclude that the sleep changes observed here depend on a global use-dependent recovery phenomenon going on during sleep, it is possible to hypothesize that they rely to some degree on the activation of specific learning-related processes. Still, this hypothesis should be further explored through a comparison with a wake condition and a greater sample size, allowing for sufficient variance to analyze correlations between sleep features and memory variables. 

As for our second research question, individual levels of expertise appear not to affect learning-dependent sleep changes, considering that most of our sleep variables were influenced by the experimental condition (BL vs. TR) but not by professional status (actors vs. non-actors) and that no interaction between these factors emerged. This negative result, along with our finding that performance did not differ between groups, is in contrast with several studies in which “good learners” (i.e., high-performing subjects at baseline assessment) showed either the greater post-sleep performance benefits [22,23] or more pronounced post-learning sleep changes [24,25]. In fact, although immediate recall performance was similar between groups (at variance with the latter studies), the actors’ higher skill and familiarity with the task might have determined a different processing of the learning material directly during sleep, accompanied by the correspondingly enhanced sleep changes. However, the understanding of the complex interplay among the factors affecting sleep-dependent memory processing (such as intentionality, awareness, task difficulty and memory trace strength) is still far from complete [13]. In this sense, our findings suggest that other factors besides expertise with the task are at play. For instance, as we have previously argued [13,51], it is plausible that the “tagging” of memories for later sleep-related processing depends on their relevance as a guide for future behavior rather than on their strength (see, e.g., [52,53]). 

An interesting remark concerns our findings on sleep onset latency. The interaction effect observed for this parameter indicates that sleep propensity was enhanced by training and that this effect was more pronounced in the group of actors (although note that its significance did not survive Bonferroni’s correction). This result is consistent with a previous study from our group, in which we showed that sleep latency was reduced in healthy adults after training for a complex task based on procedural and executive components [11]. Together with other results showing no detrimental effect of pre-sleep training on sleep latency [12], these data further support the hypothesis that sleep-related learning mechanisms are able to counteract possible arousal effects linked to the task, thus improving both sleep propensity and sleep maintenance [11,12]. Actors are likely to be less aroused by a task they are more familiar with than non-actors, and this could account for the even more pronounced reduction of their post-training sleep latency compared to the other group. 

A final comment regards performance measures. Here, again, we did not observe differences between groups. Interestingly, while scores at classical verbal tasks (i.e., mostly word lists) are usually lower at delayed compared to immediate recall (e.g., [16]), we found, instead, that performance was globally maintained at morning re-test. This is probably linked to the ecological nature of the task (relying on prose memory), which promotes recall of the semantic gist rather than of the verbatim trace. Actually, the finding that synonyms were the only performance measure to differ between the two recall phases, with a significant increase at delayed recall, is coherent with this interpretation. Furthermore, the latter finding is in line with recent views on sleep-related memory processing, which propose that the role of sleep for memory consists in trace transformation for adaptive purposes rather than rote consolidation (see [13,54] for a review). 

A few limitations of the study should be acknowledged. First, our limited sample size imposes caution in interpreting these findings. Specifically, while the effect of the learning session on sleep variables appears quite robust (it emerges from within-group rather than between-group differences in sleep parameters), negative findings on the influence of expertise were obtained, instead, from the comparison between smaller samples. Moreover, this study lacks control on some factors which could have influenced sleep features, such as the timing of meals and of caffeine and alcohol consumption. Finally, a few other variables, such as the timing of sleep periods and physical activity levels, which were kept constant within groups, could have instead affected between-groups comparisons. Nevertheless, it is worth noting that the lack of a strict control on these factors, which would have been possible in a laboratory setting, depends on our methodological choice of keeping the experimental protocol as less disrupting as possible of the participants’ everyday routines, in order to maximize ecological validity. 

## 5. Conclusions

In conclusion, our results show that the bedtime administration of a verbal ecological task improves objective sleep quality in terms of sleep continuity, stability, and cyclic organization. These findings add to previous literature suggesting that everyday-life learning processes contribute to sleep regulation and, from an applicative perspective, encourage one to consider ecological pre-sleep training sessions as a feasible approach to improve sleep quality. Moreover, while our data do not support the role of expertise as a modulating factor in sleep-related learning processes, they are in agreement with recent literature highlighting the role of sleep in the qualitative transformation of information rather than mere stabilization or enhancement, with the purpose of optimizing learning for future behavior [13,54]. 

## Figures and Tables

**Table 1:** Classical sleep measures in the two groups in BL and TR.

Descriptives				Statistics			
	Group	Condition	m ± sd	Effects	F	*p*	η^2^
Sleep Onset Latency (min)	Actors	BL	11.4 ± 8.05	Condition	60.87	**<0.001**	0.195
		TR	5.7 ± 4.03	Interaction	5.08	*0.036*	0.016
	non-Actors	BL	20.7 ± 11	Group	5.08	*0.036*	0.147
		TR	10.3 ± 5.51				
Time in Bed (h)	Actors	BL	7.55 ± 1.39	Condition	0.23	0.630	0.004
		TR	7.38 ± 0.97	Interaction	0.01	0.915	0.000
	non-Actors	BL	7.36 ± 1.27	Group	0.17	0.682	0.005
		TR	7.25 ± 0.94				
Total Sleep Time (h)	Actors	BL	7.08 ± 1.43	Condition	0.018	0.892	0.000
		TR	7.03 ± 0.93	Interaction	0.085	0.774	0.001
	non-Actors	BL	6.70 ± 1.36	Group	0.541	0.471	0.017
		TR	6.82 ± 0.82				
Actual Sleep Time (h)	Actors	BL	6.79 ± 1.47	Condition	0.577	0.456	0.010
		TR	6.89 ± 10	Interaction	0.194	0.665	0.003
	non-Actors	BL	6.38 ± 1.54	Group	0.527	0.527	0.013
		TR	6.75 ± 0.78				
Stage1 (%)	Actors	BL	22.6 ± 7.61	Condition	1.10	0.305	0.020
		TR	21.1 ± 9.23	Interaction	0.319	0.579	0.006
	non-Actors	BL	32.4 ± 14.7	Group	4.90	*0.039*	0.120
		TR	27.4 ± 12.1				
Stage2 (%)	Actors	BL	45 ± 8.43	Condition	0.112	0.742	0.002
		TR	46 ± 6.51	Interaction	0.510	0.483	0.010
	non-Actors	BL	42.6 ± 10.1	Group	2.14	0.159	0.056
		TR	39.9 ± 10.7				
SWS (%)	Actors	BL	13.6 ± 6.64	Condition	0.955	0.797	0.001
		TR	14.3 ± 3.46	Interaction	0.1399	0.483	0.010
	non-Actors	BL	11.2 ± 5.89	Group	4.09	0.057	0.098
		TR	9.58 ± 5.99				
REM (%)	Actors	BL	18.2 ± 6.8	Condition	1.48	0.237	0.024
		TR	19 ± 3.94	Interaction	0.450	0.510	0.007
	non-Actors	BL	19.4 ± 4.44	Group	0.281	0.281	0.038
		TR	22.1 ± 6.57				
Wake After Sleep Onset (%)	Actors	BL	4.2 ± 4.06	Condition	9.13	**0.007**	0.127
		TR	2.1 ± 3.87	Interaction	1.28	0.272	0.018
	non-Actors	BL	5.55 ± 7.06	Group	0.004	0.948	0.000
		TR	0.95 ± 1.4				
Sleep Efficiency	Actors	BL	89.8 ± 7.92	Condition	8.54	**0.008**	0.121
		TR	93.5 ± 6.78	Interaction	0.959	0.339	0.014
	non-Actors	BL	85.9 ± 10.8	Group	0.534	0.473	0.015
		TR	93.4 ± 4.23				

Notes. Significant results are in bold, whereas trends to significance are indicated with italics.

**Table 2:** Sleep continuity measures in the two groups in BL and TR.

Descriptives				Statistics			
	Group	Condition	m ± sd	Effects	F	*p*	η^2^
Total Awakenings frequency	Actors	BL	0.64 ± 0.51	Condition	19.94	**<0.001**	0.231
		TR	0.18 ± 0.23	Interaction	0.69	0.413	0.008
	non-Actors	BL	0.51 ± 0.42	Group	0.146	0.706	0.004
		TR	0.20 ± 0.21				
Brief Awakenings frequency	Actors	BL	0.28 ± 0.24	Condition	6.65	*0.018*	0.132
		TR	0.07 ± 0.11	Interaction	0.097	0.335	0.019
	non-Actors	BL	0.19 ± 0.25	Group	0.290	0.596	0.006
		TR	0.10 ± 0.15				
Long Awakenings frequency	Actors	BL	0.36 ± 0.30	Condition	18.50	**<0.001**	0.209
		TR	0.12 ± 0.20	Interaction	0.001	0.967	0.000
	non-Actors	BL	0.32 ± 0.28	Group	0.206	0.654	0.006
		TR	0.08 ± 0.12				
Awakenings mean duration (min)	Actors	BL	3.76 ± 3.33	Condition	3.36	0.082	0.068
		TR	3.39 ± 4.89	Interaction	2.29	0.146	0.047
	non-Actors	BL	5.47 ± 5.13	Group	0.001	0.972	0.000
		TR	1.58 ± 1.86				

Notes. Significant results are in bold, whereas trends to significance are indicated with italics. Frequency of awakenings is calculated over hours of Actual Sleep Time.

**Table 3:** Sleep stability measures in the two groups in BL and TR.

Descriptives				Statistics			
	Group	Condition	m ± sd	Effects	F	*p*	η^2^
Arousals frequency	Actors	BL	7.49 ± 2.81	Condition	43.84	**<0.001**	0.482
		TR	3.55 ± 1.46	Interaction	0.040	0.842	0.000
	non-Actors	BL	7.77 ± 2.39	Group	0.048	0.828	0.001
		TR	3.59 ± 1.94				
State Transitions frequency	Actors	BL	16.2 ± 5.04	Condition	42.98	**<0.001**	0.509
		TR	8.31 ± 2.84	Interaction	0.001	0.972	0.001
	non-Actors	BL	16.1 ± 4.17	Group	0.007	0.932	0.000
		TR	8.25 ± 3.86				
FU periods frequency	Actors	BL	1.04 ± 0.62	Condition	40.1	**<0.001**	0.485
		TR	0.20 ± 0.23	Interaction	0.006	0.980	0.000
	non-Actors	BL	1.11 ± 0.51	Group	0.271	0.608	0.004
		TR	0.28 ± 0.30				
TFU (%)	Actors	BL	9.12 ± 5.72	Condition	36.6	**<0.001**	0.470
		TR	1.47 ± 1.79	Interaction	0.009	0.976	0.000
	non-Actors	BL	9.79 ± 5.53	Group	0.301	0.589	0.004
		TR	2.21 ± 2.23				
FU periods mean duration (min)	Actors	BL	5.12 ± 0.96	Condition	13.24	**0.001**	0.243
		TR	2.96 ± 1.95	Interaction	0.375	0.547	0.007
	non-Actors	BL	5.01 ± 0.86	Group	0.165	0.689	0.003
		TR	3.48 ± 2.46				

Notes. Significant results are in bold. Arousals frequency is calculated over hours of Actual Sleep Time, while the frequency of state transitions and FU periods is computed over hours of Total Sleep Time. FU: Functional Uncertainty; TFU: Total Time spent in Functional Uncertainty.

**Table 4:** Sleep organization measures in the two groups in BL and TR.

Descriptives				Statistics			
	Groups	Condition	m ± sd	Effects	F	*p*	η^2^
number of Cycles	Actors	BL	2.27 ± 1.42	Condition	6.32	*0.021*	0.078
		TR	3.09 ± 1.22	Interaction	0.253	0.621	0.003
	non-Actors	BL	2.36 ± 1.29	Group	0.010	0.920	0.000
		TR	2.91 ± 0.94				
TCT (%)	Actors	BL	46.7 ± 19.9	Condition	6.60	*0.018*	0.152
		TR	65 ± 21.9	Interaction	0.007	0.993	0.000
	non-Actors	BL	51.9 ± 28.1	Group	0.642	0.432	0.012
		TR	70.1 ± 18.6				
Cycles mean duration (min)	Actors	BL	91.4 ± 18.8	Condition	2.08	0.165	0.053
		TR	91.7 ± 16.8	Interaction	1.96	0.177	0.050
	non-Actors	BL	81.3 ± 33.7	Group	0.009	0.976	0.000
		TR	102 ± 17.1				

Notes. Trends to significance are in italics. TCT: Total time spent in sleep cycles.

**Table 5:** Performance measures in the two groups at immediate and delayed recall.

Descriptives				Statistics			
	Groups	Recall Phase	m ± sd	Effects	F	*p*	η^2^
Global Accuracy	Actors	immediate	188 ± 31.3	Recall phase	0.060	0.808	0.000
		delayed	182 ± 30	Interaction	1.70	0.206	0.004
	non-Actors	immediate	167 ± 38.9	Group	1.39	0.252	0.061
		delayed	170 ± 34.8				
Synonyms	Actors	immediate	11.1 ± 9.19	Recall phase	15.02	**<0.001**	0.056
		delayed	16.9 ± 12.8	Interaction	2.30	0.145	0.009
	non-Actors	immediate	8.09 ± 4.74	Group	1.76	0.200	0.069
		delayed	10.6 ± 4.90				
Inversions	Actors	immediate	0.81 ± 0.98	Recall phase	0.346	0.563	0.006
		delayed	1.09 ± 1.76	Interaction	1.05	0.316	0.020
	non-Actors	immediate	2.09 ± 3.94	Group	0.674	0.421	0.020
		delayed	1.09 ± 1.45				
Omissions	Actors	immediate	19 ± 17.1	Recall phase	0.005	0.944	0.000
		delayed	21.5 ± 32.8	Interaction	1.14	0.298	0.004
	non-Actors	immediate	35.7 ± 26.5	Group	2.59	0.123	0.106
		delayed	32.8 ± 22.3				
Intrusions	Actors	immediate	4.55 ± 4.91	Recall phase	0.525	0.477	0.004
		delayed	4.09 ± 5.72	Interaction	1.76	0.199	0.015
	non-Actors	immediate	2 ± 2.45	Group	0.907	0.352	0.035
		delayed	3.55 ± 2.77				
Errors	Actors	immediate	1.18 ± 1.40	Recall phase	3.63	0.071	0.052
		delayed	2.36 ± 2.87	Interaction	0.327	0.574	0.005
	non-Actors	immediate	2 ± 1.61	Group	0.692	0.451	0.019
		delayed	2.64 ± 1.80				

Notes. Significant results are in bold.

## Data Availability

The data presented in this study are available on request from the corresponding author. The data are not publicly available due to privacy reasons.
